# The Oxidative Stress-Induced miR-200c Is Upregulated in Psoriasis and Correlates with Disease Severity and Determinants of Cardiovascular Risk

**DOI:** 10.1155/2019/8061901

**Published:** 2019-12-19

**Authors:** A. Magenta, M. D'Agostino, S. Sileno, L. Di Vito, C. Uras, D. Abeni, F. Martino, F. Barillà, S. Madonna, C. Albanesi, M. Napolitano, M. C. Capogrossi, G. Melillo

**Affiliations:** ^1^Experimental Immunology Laboratory, IDI-IRCCS, Rome, Italy; ^2^Unit of Cardiology, IDI-IRCCS, Rome, Italy; ^3^Clinical Epidemiology Unit, IDI-IRCCS, Rome, Italy; ^4^Department of Pediatrics, Sapienza University of Rome, Italy; ^5^Department of Cardiovascular, Respiratory, Nephrology, Anesthesiology and Geriatric Sciences, Sapienza University of Rome, Italy; ^6^Division of Cardiology, Johns Hopkins University, Johns Hopkins Bayview Medical Center, Baltimore, MD, USA; ^7^Laboratory of Cardiovascular Science, National Institute on Aging (NIA), National Institutes of Health (NIH), Baltimore, MD, USA

## Abstract

Psoriasis is a chronic inflammatory skin disease associated with reactive oxygen species (ROS) increase and a higher risk of cardiovascular (CV) events. We previously showed that the miR-200 family (miR-200s) is induced by ROS, miR-200c being the most upregulated member responsible for apoptosis, senescence, ROS increase, and nitric oxide decrease, finally causing endothelial dysfunction. Moreover, circulating miR-200c increases in familial hypercholesterolemic children and in plaques and plasma of atherosclerotic patients, two pathologies associated with increased ROS. Given miR-200s' role in endothelial dysfunction, ROS, and inflammation, we hypothesized that miR-200s were modulated in lesional skin (LS) and plasma of psoriatic patients (Pso) and that their levels correlated with some CV risk determinants at a subclinical level. All Pso had severe psoriasis, i.e., Psoriasis Area and Severity Index (PASI) > 10, and one of the following: at least two systemic psoriasis treatments, age at onset < 40 years, and disease duration > 10 years. RNA was extracted from plasma (Pso, *N* = 29; Ctrl, *N* = 29) and from nonlesional skin (NLS) and LS of 6 Pso and 6 healthy subject skin (HS) biopsies. miR-200 levels were assayed by quantitative RT-PCR. We found that all miR-200s were increased in LS vs. NLS and miR-200c was the most expressed and upregulated in LS vs. HS. In addition, circulating miR-200c and miR-200a were upregulated in Pso vs. Ctrl. Further, miR-200c positively correlated with PASI, disease duration, left ventricular (LV) mass, LV relative wall thickness (RWT), and *E*/*e*′, a marker of diastolic dysfunction. Multiple regression analysis indicates a direct association between miR-200c and both RWT and LV mass. Circulating miR-200a correlated positively only with LV mass and arterial pressure augmentation index, a measure of stiffness, although the correlations were nearly significant (*P* = 0.06). In conclusion, miR-200c is upregulated in LS and plasma of Pso, suggesting its role in ROS increase and inflammation associated with CV risk in psoriasis.

## 1. Introduction

Psoriasis is a chronic inflammatory disease in which both genetic and environmental factors play an important pathogenic role. Psoriasis presents, in most cases, with a chronic plaque form and can be associated with several comorbidities, such as metabolic syndrome, kidney or gastrointestinal diseases, diabetes, cardiovascular diseases (CVD), and cerebrovascular diseases (CeVD) [1, 2].

Although early concepts of psoriasis pathogenesis focused primarily on keratinocyte hyperproliferation, the current view identifies dysregulation of immune responses and systemic inflammation as key pathogenic events [1]; moreover, an increase in reactive oxygen species production and decreased antioxidant potential in psoriasis have been also described [3, 4]. As a result, psoriasis is now considered a systemic disease in which oxidative stress and inflammation are widespread [3–6]. Importantly, life expectancy is reduced in severe psoriasis by up to 4 years, largely because of the high incidence of CVD and CeVD [6–8].

It is still debated whether psoriasis may be regarded as an independent risk factor for CVD [9], as patients with severe psoriasis have a higher prevalence of traditional cardiovascular (CV) risk factors, including smoking, dyslipidemia, diabetes, and hypertension [7, 10, 11]. On the other hand, systemic inflammation may induce atherosclerosis progression [12] and in severe psoriasis is associated with an elevated production of oxygen free radicals, which in turn induce vascular endothelial cell (EC) damage.

Endothelial dysfunction is an early sign of atherosclerosis, because it represents the initial stage of atherosclerotic plaque formation, and is associated with altered gene expression that promotes inflammatory cell adhesion and lipid deposition within the intima and media layers of the vascular wall. Endothelial cell dysfunction can be measured, indirectly, by assessing the brachial artery flow-mediated dilatation, which can be associated with the inability of a dysfunctional endothelium to release nitric oxide (NO) [13]. An advanced sign of vascular damage is represented by increased arterial stiffness [14].

A previous study from our group demonstrated that psoriatic patients at high CV risk show an increase in cardiac hypertrophy and left ventricular (LV) remodeling, as well as an increase in arterial stiffness, when compared to patients without psoriasis and high CV risk. These data show that psoriasis may exert an additional negative effect on the CV system in patients with a high prevalence of traditional CV risk factors [15].

MicroRNAs (miRNAs) are short noncoding RNAs that modulate the stability and the translational efficiency of target messenger RNAs (mRNA) [16]. miRNAs have been linked to different diseases, and given their stability in plasma, circulating miRNA altered expression levels have been used as noninvasive biomarkers for different diseases, including CVD [17–20].

Although different miRNAs have been linked to psoriasis [21–23], little is known on miRNAs associated with CVD increase in this disease.

We previously showed that the miR-200 family (i.e., miR-200a, miR-200b, miR-200c, miR-141, and miR-429) is induced by reactive oxygen species (ROS) in ECs [24].

Among miR-200 family members, miR-200c exhibited the highest ROS-induced upregulation in ECs and it is also upregulated in skin fibroblasts and in muscle cells, upon oxidative stress exposure [24]. The miR-200c increase was responsible for EC apoptosis, senescence, and dysfunction [24, 25]. At the molecular level, miR-200c was shown to directly target Sirtuin1 (SIRT1), endothelial nitric oxide synthase (eNOS), and the transcription factor forkhead box O1 (FOXO1), which are involved in EC function preservation [25]. Thus, miR-200c upregulation causes ROS increase and NO production decrease [25]. Moreover, we have previously shown that circulating miR-200c is significantly upregulated in familial hypercholesterolemia (FH) patients in pediatric age [26], a disease associated with high oxidative stress levels and early atherosclerosis [27, 28].

Recently, we found that miR-200c is upregulated in carotid plaques and its expression is higher in unstable vs. stable plaque, its expression levels correlated with inflammatory markers, and circulating miR-200c levels were highly upregulated in the plasma of atherosclerotic patients compared to healthy subjects [29].

Notably, proinflammatory responses in vascular cells play a fundamental role in atherosclerosis development [30, 31].

Therefore, given the role of the miR-200 family (miR-200s) in oxidative stress, endothelial dysfunction, and inflammation, all features associated with psoriasis, we wondered whether the miR-200 family was modulated in skin and in plasma of psoriatic patients and whether miR-200 family member levels correlated with CV risk associated with this disease.

## 2. Materials and Methods

### 2.1. Subject Selection and Classification

Patients affected by chronic plaque psoriasis (Pso, *N* = 29) and controls (Ctrl, *N* = 29) were prospectively enrolled from 2014 to 2016 at the Istituto Dermopatico dell'Immacolata- (IDI-) IRCCS, Rome, Italy. All patients, afferent to the Integrated Research Centre for Psoriasis of IDI-IRCCS, had severe psoriasis defined by the Psoriasis Area and Severity Index (PASI) greater than 10 and at least one of the following conditions: previous history of at least two systemic treatments for psoriasis, psoriasis onset before 40 years of age, and psoriasis disease duration of more than 10 years.

Exclusion criteria were diabetes, history of cerebrovascular events, myocardial infarction and/or myocardial revascularization, and psoriatic arthritis. Additional exclusion criteria were positivity for hepatitis B (HBsAg, anti-HBc), hepatitis C (anti-HCV), human immunodeficiency virus (anti-HIV 1 and 2); presence of a cancer or of a haematological disease; pregnancy or breastfeeding; neuropsychiatric diseases interfering with the patient's collaboration to the study; radiotherapy; chemotherapy; or systemic treatment with corticosteroids or immunosuppressive agents.

Anthropometric data, including gender, weight, height, and body mass index (BMI), were measured for all subjects. Clinical features were also recorded, including disease duration, medications, blood pressure, and smoking habit, as shown in Table 1.

The study was approved by the Ethic Committee of IDI-IRCCS (Prot. no. 188/CE/2014 approved in 19/09/2014), and all patients provided written informed consent at enrollment. Skin biopsies were obtained from 6 healthy subjects and 6 Pso patients from lesional (LS) and nonlesional (NLS) areas of the same patient. Two different studies approved by the Ethic Committee of IDI-IRCCS were used (Prot. no. 34/CE/2016 approved in 14/10/2016 and Prot. no. 47/CE/2016 approved in 18/11/2016, respectively). All patients provided written informed consent at enrollment.

The study was conducted following the Good Clinical Practice guidelines and according to the declaration of Helsinki.

### 2.2. Transthoracic Echocardiography

A single experienced cardiologist performed in all patients a comprehensive two-dimensional (2D) transthoracic echocardiographic examination using a commercially available device (Vivid 7, GE Vingmed Ultrasound AS, Horten, Norway). Data acquisition was performed with a 2.5-3.5 MHz transducer in the parasternal and apical views (standard parasternal long-axis and short-axis, apical, four-chamber, and two-chamber views). Pulsed-wave and continuous-wave Doppler analysis of flow velocities, as well as Color Doppler and Tissue Doppler analysis, was routinely used to obtain echocardiographic parameters according to the American Society of Echocardiography standards [32]. The LV mass index was calculated as the ratio between the LV mass and body surface area (BSA).

Relative wall thickness (RWT) was calculated at the ventricular diastole by dividing the sum of septum wall thickness and posterior wall thickness by the left ventricle internal diameter. Patterns of LV remodeling or hypertrophy were defined by echocardiography, on the basis of relative wall thickness (RWT) (cutoff value 0.42) and LV mass index (cutoff 95 gr/m^2^ in female subjects and 115 gr/m^2^ in male subjects).

Echocardiographic parameters are summarized in Table 2.

### 2.3. Blood Pressure Measurement, Wave Reflection Analysis, and Pulse Wave Velocity Calculation

Systolic and diastolic peripheral (brachial) pressure measurement, wave reflection analysis, and pulse wave velocity (PWV) calculation were performed by using a validated, commercially available system (SphygmoCor XCEL, AtCor Medical, Sydney, Australia), as previously described [33–35].

Wave reflection analysis consisted of central (aortic) pressure assessment, together with the calculation of two indirect indexes of aortic stiffness: augmentation index and augmentation pressure.

Carotid-femoral PWV, a direct index of aortic stiffness [35–37], was calculated as the distance travelled by the pulse wave divided by the time difference between the feet of carotid and femoral arterial waveforms. A PWV cutoff value greater than 8.0 m/sec was used as a marker of increased arterial stiffness in asymptomatic patients [37, 38].

Blood pressure, wave reflection analysis, and pulse wave velocity data are summarized in Table 3.

### 2.4. Plasma and Skin Samples

Plasma samples were collected from peripheral blood of 29 Pso and 29 Ctrl subjects following the standard laboratory procedures. In all subjects, blood samples were obtained from the antecubital brachial vein after an overnight fast of at least 8 h and used to measure total cholesterol, low-density lipoprotein (LDL) cholesterol, high-density lipoprotein (HDL) cholesterol, and blood glucose levels.

The clinical characteristics of these subjects are described in Table 1.

Venous blood samples (10 ml) were collected in EDTA-containing tubes. Blood was then centrifuged (1200 × *g* for 10 min at 4°C), and supernatants were collected and centrifuged (12000×*g* for 10 min at 4°C). Plasma samples were stored at −80°C and thawed on ice before use.

6 mm punch biopsies were taken from LS and NLS areas of the same Pso patient (*N* = 6) and from skin of healthy volunteers undergoing plastic surgery (*N* = 6). Pso patients were not undergoing topical treatments. Skin biopsies were immediately frozen in nitrogen liquid at -180°C.

### 2.5. RNA Extraction and Analysis

RNA extraction was performed from 200 *μ*l of plasma samples. miRNAs were isolated using Total RNA Purification Plus Kit (Norgen Biotek, Thorold, ON, Canada) according to the manufacturer's protocol, as previously described [39]. As an internal control, 10 ftmoles of cel-miR-39a was spiked into each plasma sample after lysis.

RNA from skin biopsies was extracted with an RNeasy tissue lipid kit (Qiagen, Valencia, CA, USA), as previously described [40]. miRNA expression levels in skin samples were normalized to housekeeping RNA Z30 expression.

miRNA levels were analysed using the TaqMan quantitative real-time PCR (qRT-PCR) and quantified with the QuantStudio5 real-time PCR (Thermo Fisher Scientific, Massachusetts, United States). Primers for miR-200c, miR-200a, miR-200b, miR-429, miR-141, Z30, and cel-miR-39a and the reagents for reverse transcriptase and qPCR reactions were all obtained from Applied Biosystems. miRNA expression levels in each sample were normalized to cel-miR-39a. Relative expression in fold was calculated using the comparative Ct method (2^–*ΔΔ*Ct^) [41].

Given the logarithmic nature of the q-PCR CT, a decrease in Δ*C*T corresponds to an increase in miRNA levels in the analysed samples. Therefore, data were expressed as −Δ*C*T, in order to obtain a positive correlation between miRNA levels and clinical parameter values.

### 2.6. Statistical Analyses

Because of the exploratory nature of this study, whose primary objectives are mainly descriptive, the minimum sample size has not been predetermined.

Each quantitative variable was checked for normality distribution by the D'Agostino and Pearson omnibus normality test. The Mann-Whitney, Wilcoxon rank test, or Student *t*-test were used when appropriate, as indicated in figure legends. Descriptive data are presented as absolute numbers and percentages for categorical variables and as means and standard error means (S.E.M.) for continuous variables. The groups were compared with the unpaired Student *t*-test for continuous and with Fisher's exact test for categorical variables. Correlation analyses were carried out by Spearman's test.

Statistical significance was defined at *P* < 0.05.

In order to measure the impact of different independent variables on the variable RWT and LV mass index, multivariate regression models were estimated. The regression models (see Table 4) took into account RWT (models 4a and 4b) and LV mass index (models 4c and 4d) as the dependent variables and the following variables as predictors: Pso, age, sex, and miR-200c. Models 4a and 4c took into account all the variables described above; in models 4b and 4d, we excluded the variable Pso.

All statistical analyses were performed using the statistical software packages GraphPad Prism 5.0 (GraphPad, San Diego, CA, USA) and Stata (StataCorp LLC, College Station, TX, USA).

## 3. Results

### 3.1. Clinical Characteristics of Patients

The clinical characteristics of Pso and Ctrl subjects are reported in Table 1. The two groups were homogeneous for clinical, anthropometric, and laboratory parameters with the exception of total cholesterol values that were higher in Pso vs. Ctrl, in agreement with lipid abnormalities associated with this disease [15, 42, 43]. Also, erythrocyte sedimentation rate (ESR) was higher in Pso compared to Ctrl, and ESR is a delayed inflammatory biomarker known to increase in Pso [44]. Blood pressure, wave reflection analyses, and PWV were similar in the Pso and Ctrl groups (Table 3).

Echocardiographic parameters were measured in most patients (Table 2). All patients included in this study had a normal LV ejection fraction (LVEF) (i.e., greater than 55%) and did not show regional LV wall motion abnormalities. Pso patients had an increase in LV mass index and RWT, as previously observed [15].

### 3.2. miR-200 Levels Are Increased in Lesional vs. Nonlesional Skin of Psoriatic Patients

We evaluated miR-200 family expression in nonlesional skin (NLS) and lesional skin (LS) of patients with psoriasis and in healthy control skin (HS).

**Figure 1:**
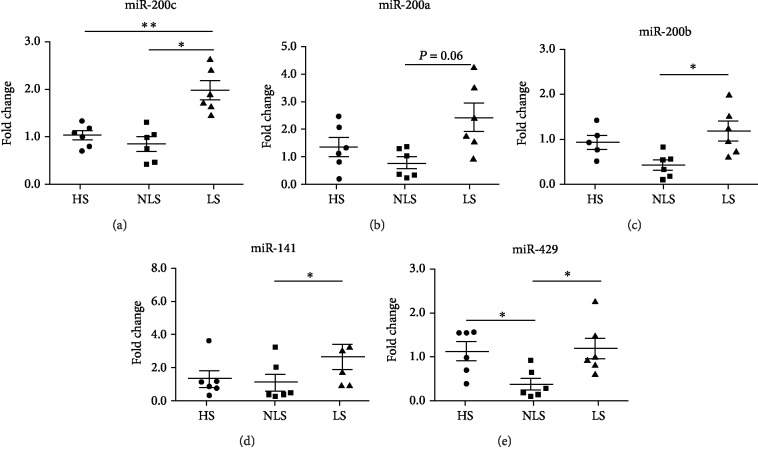
The miR-200 family increases in lesional vs. nonlesional skin of psoriatic patients. Total RNA was extracted from nonlesional skin (NLS) and lesional skin (LS) of 6 psoriatic patients and from 6 healthy control skin biopsies (HS). The RNA was assayed for miR-200c family expression by qRT-PCR. The entire miR-200 family was upregulated in LS compared to NLS. (a) miR-200c was upregulated in LS compared to HS (Mann-Whitney test; ^∗∗^*P* < 0.01) and in LS vs. NLS (Wilcoxon rank test; ^∗^*P* < 0.05). (b) miR-200a was upregulated in LS vs. NLS (*P* = 0.06). (c) miR-200b was upregulated in LS compared to NLS (Wilcoxon rank test; ^∗^*P* < 0.05). (d) miR-141 was upregulated in LS with respect to NLS (Wilcoxon rank test; ^∗^*P* < 0.05). (e) miR-429 was downregulated in NLS compared to HS (Mann-Whitney test; ^∗∗^*P* < 0.01) and upregulated in LS compared to NLS (Wilcoxon rank test; ^∗^*P* < 0.05).

To this aim, we collected skin specimens from 6 Pso and 6 HS. As expected, inflammatory biomarkers typically overexpressed in psoriatic skin were found to be upregulated in LS compared to NLS or HS areas [40]. Interestingly, we found that the entire miR-200 family was significantly upregulated in LS compared to NLS areas of the same psoriatic patient (Figure 1), although miR-200a upregulation was nearly statistically significant (Figure 1(b), *P* = 0.06).

Indeed, the most expressed miRNA in the skin was miR-200c which was also upregulated in LS vs. HS (Figure 1(a), ^∗∗^*P* < 0.01).

Notably, miR-200c and miR-141 are clustered on chromosome 12, whereas miR-200a, miR-200b, and miR-429 are clustered on chromosome 1.

Interestingly, the cotranscribed miR-200a, miR-200b, and miR-429 were slightly decreased in NL compared to HS and miR-429 significantly decreased in NL compared to HS (Figures 1(b), 1(c), and 1(e)).

Conversely, the levels of miR-200c and the cotranscribed miR-141 were similar in both NLS and HS (Figures 1(a) and 1(d)).

These findings show that miR-200 family expression levels are increased in LS vs. NLS of psoriatic patients; miR-200c is also increased in LS compared to HS.

### 3.3. Circulating miR-200c and miR-200a Are Upregulated in Plasma of Psoriatic Patients Compared to Healthy Controls

**Figure 2:**
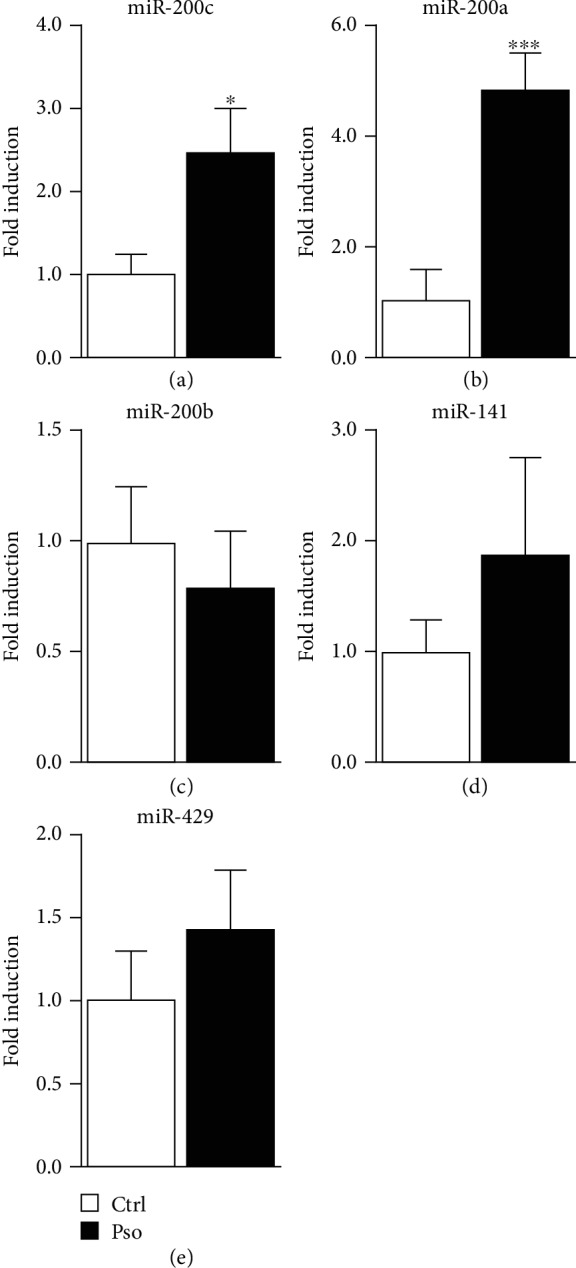
Circulating miR-200c and miR-200a levels increase in psoriatic patients. Total RNA extracted from plasma of healthy control subjects (Ctrl, *N* = 29) and psoriatic patients (Pso, *N* = 29) was assayed for miR-200c family expression by qRT-PCR. (a) Circulating miR-200c was upregulated in Pso compared to Ctrl (unpaired Student *t*-test; ^∗^*P* < 0.05). (b) Circulating miR-200a was upregulated in Pso compared to Ctrl (unpaired Student *t*-test; ^∗∗∗^*P* < 0.001). (c, d, e) Circulating miR-200b, miR-141, and miR-429 were not significantly modulated in Pso compared to Ctrl.

We analysed circulating miR-200 family expression levels in plasma of psoriatic patients (Pso, *N* = 29) compared to healthy controls (Ctrl, *N* = 29). We found that miR-200c was significantly upregulated in Pso nearly 2.5-fold compared to Ctrl (Figure 2(a), ^∗^*P* < 0.05) and that miR-200a was upregulated in Pso nearly 5-fold compared to Ctrl (Figure 2(b), ^∗∗∗^*P* < 0.001). miR-141 and miR-429 were slightly upregulated in Pso compared to Ctrl, although not significantly (Figures 2(d) and 2(e)), whereas miR-200b was not modulated (Figure 2(c)).

### 3.4. Circulating miR-200c Positively Correlates with PASI and Psoriasis Duration

**Figure 3:**
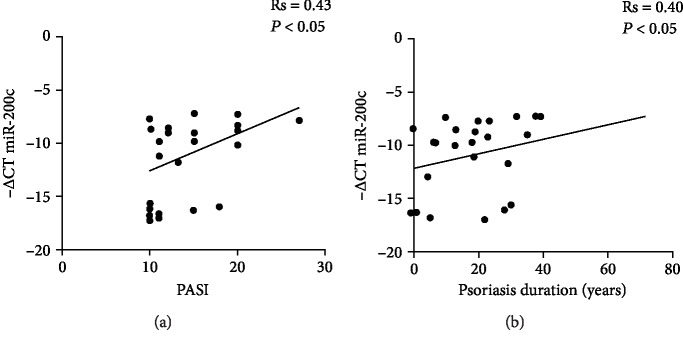
Circulating miR-200c levels positively correlate with PASI and disease duration. Correlation analyses of circulating miR-200c levels with (a) Psoriasis Area and Severity Index (PASI) and (b) the duration of disease expressed in years (Spearman's correlation test).

We next tested whether circulating miR-200c expression levels may correlate with a recognized index of psoriasis severity, i.e., PASI and duration of disease. Our results showed that miR-200c positively correlated with both PASI (Figure 3(a); Rs = 0.43, ^∗^*P* < 0.05) and duration of disease (Figure 3(b); Rs = 0.4, ^∗^*P* < 0.05). Correlation analyses of miR-200c were also performed by clinical laboratory tests shown in Table 1, but no significant correlation was found.

In contrast, circulating miR-200a expression levels did not correlate significantly with PASI and duration of disease or with the clinical laboratory test examined.

### 3.5. Circulating miR-200c Correlates with LV Remodeling and with Diastolic Dysfunction

miR-200c has been previously shown to play an important role in cardiac hypertrophy, as shown in animal models [45, 46].

Since miR-200c has a role in endothelial dysfunction [25] and increases in both plaques and plasma of atherosclerotic patients [29], we evaluated whether miR-200c expression levels may relate to echocardiographic findings associated with an increased CV risk. To this aim, we correlated circulating miR-200c levels with some echocardiographic parameters (see Table 2) assessed both in Pso patients (*N* = 17) and in Ctrl patients (*N* = 23).

**Figure 4:**
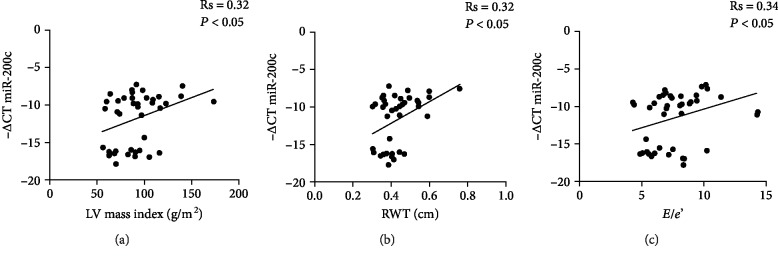
Correlation analyses of circulating miR-200c with echocardiographic parameters. Correlation of circulating miR-200c levels with (a) left ventricular (LV) mass index, (b) relative wall thickness (RWT), and (c) *E*/*e*′ parameter (Spearman's correlation test).

Interestingly, we found a positive correlation of miR-200c with the LV mass index (Figure 4(a); Rs = 0.32, ^∗^*P* < 0.05) and with RWT (Figure 4(b); Rs = 0.32, ^∗^*P* < 0.05), suggesting a correlation with LV hypertrophy. Moreover, we found a positive correlation with the *E*/*e*′ ratio (Figure 4(c); Rs = 0.34, ^∗^*P* < 0.05), an index that increases because of the loss of LV compliance to diastolic filling and is therefore a marker of LV diastolic dysfunction.

We also correlated circulating miR-200c levels with brachial artery systolic pressure and diastolic pressure and with wave reflection analyses (Table 3; *N* = 29 Pso and *N* = 29 Ctrl). Although the correlations were positive, we failed to find statistically significant values with systolic pressure, diastolic pressure, central systolic pressure, central diastolic pressure, central pulse pressure, augmentation pressure, augmentation index, and pulse wave velocity (PWV).

We then performed multivariate regression analyses for RWT and LV mass index, and the outcomes are reported in Table 4. The results of model 4a indicated that Pso and miR-200c were statistically significant positive-signed regressors for RWT, indicating a positive relationship with miR-200c expression levels (*P* < 0.05 for model 4a). If we excluded the Pso variable from the regression analysis (model 4b), the significant predictors of the dependent variable RWT were age and miR-200c (*P* < 0.01, Table 4), all of them positive signed.

The results of model 4c indicated that Pso was statistically significant positive-signed regressor for the LV mass index (*P* < 0.05 for model 4c); a positive relationship with miRNA expression levels was present although not significant (*P* = 0.079 for model 4c). If we excluded the Pso variable from the regression analysis (model 4d), the significant predictors of the dependent variable LV mass was miR-200c (*P* < 0.05, Table 4), which is positively signed.

Since miR-200a was also found to be upregulated in cardiomyocyte hypertrophy [47], we wondered whether its circulating levels correlated with CV risk.

**Figure 5:**
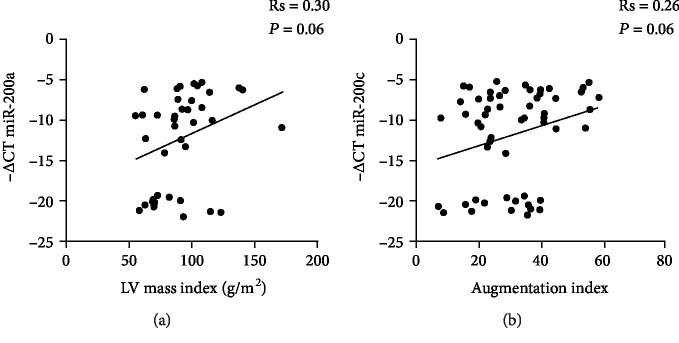
Correlation analyses of circulating miR-200a with CV risk parameters. Correlation of circulating miR-200a levels with (a) left ventricular (LV) mass index (Spearman's correlation test) and (b) augmentation index (Spearman's correlation test).

We therefore analysed whether circulating miR-200a levels correlate with the echocardiographic parameters (Table 2) evaluated in Pso patients (*N* = 17) and in Ctrl patients (*N* = 23). We found a positive correlation of circulating miR-200a levels only with the LV mass index that almost reached statistical significance (Figure 5(a); Rs = 0.3, *P* = 0.06), different from the other parameters analysed. Further, circulating miR-200a levels correlated positively with the augmentation index, which is a measure of large central artery stiffness (Figure 5(b); Rs = 0.26, *P* = 0.06), but showed no correlation with brachial artery systolic pressure and diastolic pressure and with wave reflection analyses (Table 3; *N* = 29 Pso and *N* = 29 Ctrl).

In conclusion, circulating miR-200c correlates with LV remodeling, hypertrophy, and diastolic dysfunction. Circulating miR-200a shows a positive correlation with LV hypertrophy and central arterial stiffness, although it did not reach statistical significance.

## 4. Discussion

Our observations are in agreement with previous studies reporting an increase in subclinical atherosclerosis in Pso patients when compared to normal subjects [48–51].

Psoriasis has been associated with echocardiographic abnormalities. In a study by Biyik et al., a significant increase in cardiac abnormalities such as LV hypertrophy and LV diastolic dysfunction in Pso patients was found [23]. Moreover, diastolic dysfunction has been reported in Pso patients despite a profile of low cardiovascular risk [52].

Our results show that the miR-200 family is induced in lesional vs. nonlesional skin in patients with psoriasis; moreover, miR-200c is the most expressed in skin biopsies and is also upregulated compared to that in healthy skin.

These data support a possible role of miR-200c and possibly of other members of the family in the senescent phenotype associated with psoriatic keratinocytes, given the well-known role of miR-200c in the establishment of apoptosis and senescence [24, 25]. Moreover, miR-200c and miR-200a both target SIRT1 [25, 53], a key protein involved in oxidative stress and skin inflammation regulation and in the preservation of different biological processes substantially altered in psoriasis, such as proliferation, differentiation, and senescence [4, 54]. Interestingly, SIRT1 expression is downregulated in psoriatic skin, especially in the epidermal compartment [4], and it has been shown that SIRT1 demise is associated with increased oxidative stress-induced apoptosis in Pso fibroblasts [4]. Most probably, SIRT1 decrease could be dependent on miR-200c and miR-200a local overexpression.

Further, miR-200c and miR-200a were significantly induced in the plasma of psoriatic patients vs. healthy subjects.

The miR-200 family was shown to be induced by oxidative stress and to play an important role in endothelial function; in particular, miR-200c induces EC apoptosis, senescence, and dysfunction [24, 25]. Moreover, circulating miR-200c is upregulated in FH in pediatric age, a disease characterized by oxidative stress increase and precocious atherosclerosis [26]. miR-200c increases also in carotid plaques, and its expression is higher in unstable vs. stable plaques and is upregulated in the plasma of atherosclerotic patients. Further, miR-200c expression displays a positive correlation with the inflammatory molecules interleukin 6 (IL-6) and cyclooxygenase 2 (COX-2) and also with plaque destabilizators, such as metalloproteinase- (MMP-) 1 and MMP-9 [29].

Inflammatory responses in vascular smooth muscle cells (VSMCs) are known to elicit atherosclerosis [30]. A link between the miR-200 family and inflammation has been demonstrated in VSMCs of diabetic mice [31], where an increase in the miR-200 family induces inflammatory responses through the upregulation of the monocyte chemoattractant protein 1 (MCP-1) and of COX-2 [31].

Notably, psoriasis shares striking similarities with atherosclerosis. The typical histological features of the psoriatic plaque with inflammation and leucocyte infiltration are similar to those of the atherosclerotic plaque. Further, in both atherosclerosis and psoriasis, the activation of the innate immune system initiates an inflammatory cascade that involves T lymphocytes and inflammatory cytokines that may lead to atherosclerotic plaque formation and destabilization [12]. In keeping, psoriasis shows increased CV morbidity caused by multiple factors, including systemic inflammation, oxidative stress, altered lipid profile, and concomitant established risk factors [55].

Our previous study demonstrated that psoriasis patients at high CV risk show an increase in RWT and LV mass index and PWV compared to nonpsoriatic controls; thus, psoriasis patients show an increase in cardiac hypertrophy and LV remodeling, as well as an increase in arterial stiffness. These data suggest that psoriasis may exert an additional negative effect on the CV system in patients with a higher prevalence of traditional CV risk factors [15]. Furthermore, medial-intimal carotid thickness (cIMT) is an index of atherosclerosis and is associated with the presence of multiple CV risk factors [56, 57]. An increase in cIMT in psoriasis patients compared to controls has been already reported, and a positive correlation of cIMT with age, duration of the disease, and severity of psoriasis was shown [58].

In this report, we found a statistically significant correlation of miR-200c with PASI and duration of disease; thus, miR-200c seems to correlate with the severity of disease and chronic inflammation.

Moreover, miR-200c and miR-200a have been recently associated with cardiac hypertrophy in animal models [45–47]; further, both miR-200c and miR-200a target SIRT1 [25, 53], and in addition, we demonstrated that miR-200c targets eNOS, contributing to endothelial dysfunction establishment increasing ROS and decreasing NO, that is a potent vasodilator necessary for endothelial function preservation [59]. Moreover, miR-200c decreases also the transcription factor forkhead box O1 (FOXO1) that is necessary for catalase and manganese superoxide dismutase transcription (MnSOD), further decreasing the antioxidant capacity of the cell increasing ROS production [24, 25].

In light of these observations, we examined whether these miRNAs correlated with indicators of enhanced CV risk in psoriasis.

We found that miR-200c correlated with the LV mass index and RWT, two indicators of LV remodeling and hypertrophy. Moreover, miR-200c positively correlated with the *E*/*e*′ ratio, an index of diastolic LV function.

An *E*/*e*′ ratio increase is a measure of LV relaxation abnormality and thus of LV diastolic dysfunction. Moreover, regression analyses revealed that both RWT and LV mass index were positively affected by miR-200c, indicating that besides psoriasis, a direct relationship of miR-200c in RWT and LV mass increase does exist.

The results of the present work on miR-200c and cardiac mass and function are in agreement with those of a prior study from our group on miR-200c expression in the LV of a mouse model of cardiomyopathy, induced by the anthracycline doxorubicin (DOX) [60]. We found that DOX treatment increased LV dysfunction and induced miR-200c expression compared to the control group, and chemokine SDF1 treatment partially reverted the adverse remodeling and decrease DOXO-induced miR-200c upregulation in mouse hearts [60].

Taken together, the results of the present work and our prior study suggest that the increase in miR-200c expression levels in the heart is associated with heart adverse remodeling.

On the other hand, we found that miR-200a correlated with the LV mass index and augmentation index, albeit nearly significant, and thus correlates with cardiac hypertrophy and with arterial stiffness, both important determinants of CV risk. The augmentation index, in fact, is a measure of systemic arterial stiffness derived from the ascending aortic pressure waveform.

Both miR-200c and miR-200a expression level modulations were previously linked to psoriasis. In particular, miR-200c was shown to decrease in peripheral blood mononuclear cells of psoriasis patients compared to control [61], and miR-200a was shown to be upregulated in CD4+ T cells, causing immune dysfunction through Th17/Treg cells and relevant cytokines in psoriasis vulgaris patients [62].

Our results show an increase in both miRNAs in lesional skin and in plasma of patients with psoriasis, although we cannot rule out different modulations of miR-200c in PBMNCs, since we did not analyse miR-200c expression in PBMCs of psoriasis patients. A miR-200c decrease in PBMCs could represent a compensatory mechanism. Thus, the increase in circulating miR-200c and miR-200a could be ascribed to proteins that bind to microRNAs, exosomes, microvesicles, or apoptotic bodies released from psoriatic lesions or other tissues, probably induced by the oxidative stress and inflammation that characterize this disease.

Limitations of the present study are the small number of patients and controls analysed and the fact that all patients had severe psoriasis. It will be interesting to evaluate the expression levels of miR-200s in mild to moderate psoriasis in future studies. Moreover, since most patients were treated, we could not rule out the effect of therapeutic treatments on miRNA expression levels or on CV risk parameters here evaluated.

## 5. Conclusions

Our results showed that all miR-200s are upregulated in psoriatic skin lesions. Moreover, circulating miR-200c and miR-200a are upregulated in psoriasis, and miR-200c correlates with disease severity, as indicated by PASI and duration. Further, circulating miR-200c correlates with cardiac hypertrophy and diastolic dysfunction as assessed by *E*/*e*′ and miR-200a with cardiac hypertrophy and arterial stiffness. In light of the findings of the present work and of the previously described negative effects of miR-200c and miR-200a on endothelial [24, 25, 29, 63] and vascular smooth muscle cells [31] and cardiac [45–47] function, the upregulation of these miRNAs could play a role in the inflammation and oxidative stress upregulation, increasing the risk of adverse CV events in psoriatic patients.

## Figures and Tables

**Table 1:** Baseline clinical and laboratory parameters.

Characteristic	Control healthy subject (*n* = 29)	Psoriatic subject (*n* = 29)	Comparison between two groups (*P* value)
Male sex, *N* (%)	19 (56)	15 (52)	0.42
Age (years)	49 ± 2	49 ± 3	0.95
BMI (kg/m^2^)	27 ± 1	29 ± 1	0.33
Waist circumference (cm)	103 ± 3	104 ± 3	0.85
Blood glucose (mg/dl)	96 ± 2	99 ± 7	0.68
Total cholesterol (mgl/dl)	193 ± 6	213 ± 7	0.04^∗^
LDL-cholesterol (mgl/dl)	120 ± 6	127 ± 9	0.53
HDL-cholesterol (mgl/dl)	52 ± 2	54 ± 2	0.66
Triacylglycerols (mgl/dl)	115 ± 12	108 ± 7	0.58
HsCRP (mg/l)	2 ± 1	9 ± 4	0.10
ESR (mm/h)	6 ± 2	13 ± 3	0.06
BSA (m^2^)	2 ± 0	2 ± 0	0.54
Diabetes mellitus, *N* (%)	0	0	1
Ever smoking, *N* (%)	13 (45)	10 (35)	0.59

Values are means ± S.E.M. (^∗^*P* < 0.05, between groups). Comparisons between two groups were carried out by performing the unpaired Student *t*-test for all variables with the exception of the variable “male sex,” “diabetes,” and ever smoking for which Fisher's exact test was performed. BMI: body mass index; LDL: low-density lipoprotein; hs-CRP: high-sensitivity C-reactive protein; ESR: erythrocyte sedimentation rate; BSA: body surface area.

**Table 2:** Echocardiographic parameters.

Characteristic	Control healthy subject (*n* = 23)	Psoriatic subject (*n* = 17)	Comparison between two groups (*P* value)
Male sex, *N* (%)	16 (70)	10 (59)	0.50
Age (years)	48 ± 2	50 ± 3	0.60
LV mass index (g/m^2)^	84 ± 5	103 ± 5	0.02^∗^
RWT (cm)	0.4 ± 0	0.5 ± 0	0.01^∗∗^
*E*/*e*′	7 ± 1	8 ± 0	0.80

Values are means + S.E.M. (^∗^*P* < 0.05, ^∗∗^*P* < 0.01 between groups). Comparisons between two groups were carried out by performing the unpaired Student *t*-test for all variables with the exception of the variable “male sex,” for which Fisher's exact test was performed. LV: left ventricular; RWT: relative wall thickness.

**Table 3:** Blood pressure, wave reflection analyses, and PWV.

Characteristic	Control healthy subject (*n* = 29)	Psoriatic subject (*n* = 29)	Comparison between two groups (*P* value)
Male sex, *N* (%)	19 (56)	15 (52)	0.42
Age (years)	49 ± 2	49 ± 3	0.95
Central systolic pressure (mmHg)	130 ± 3	134 ± 3	0.33
Central diastolic pressure (mmHg)	81 ± 1	82 ± 2	0.63
Central pulse pressure (mmHg)	38 ± 1	41 ± 2	0.16
Augmentation pressure (mmHg)	12 ± 1	14 ± 2	0.24
Augmentation index	30 ± 2	33 ± 3	0.40
Systolic pressure (mmHg)	120 ± 2	124 ± 3	0.25
Diastolic pressure (mmHg)	82 ± 1	83 ± 2	0.76
PWV (m/s)	7 ± 0	7 ± 0	0.91

Values are means ± S.E.M. Comparisons between two groups were carried out by performing the unpaired Student *t*-test for all variables with the exception of the variable “male sex,” for which Fisher's exact test was performed. PWV: pulse wave velocity.

**Table 4:** Multivariate regression analyses.

Model	Model 4a	Model 4b
Dependent variable	RWT	RWT
Independent variables^1^		
Psoriasis	0.054 (0.024)^∗^	—
Sex	-0.034 (0.024)	-0.039 (0.025)
Age	0.004 (0.001)^∗∗^	0.004 (0.001)^∗∗^
miR-200c	0.008 (0.003)^∗^	0.010 (0.003)^∗∗^

Significance codes (^∗^*P* < 0.05, ^∗∗^*P* < 0.01, and ^∗∗∗^*P* < 0.001). ^1^*β* coefficient and S.E. in parentheses.

## Data Availability

The data used to support the findings of this study are available from the corresponding author upon request.

## Abbreviations

BMI: Body mass index
BSA: Body surface area
CeVD: Cerebrovascular diseases
cIMT: Medial-intimal carotid thickness
COX-2: Cyclooxygenase 2
CV: Cardiovascular
CVD: Cardiovascular diseases
DOX: Doxorubicin
EC: Endothelial cell
eNOS: Endothelial nitric oxide synthase
ESR: Erythrocyte sedimentation rate
FH: Familial hypercholesterolaemia
FOXO1: Forkhead box O1
HDL: High-density lipoprotein
HS: Healthy subject skin
hs-CRP: High-sensitivity C-reactive protein
IL-6: Interleukin 6
LDL: Low-density lipoprotein
LS: Lesional skin
LVEF: LV ejection fraction
MCP-1: Monocyte chemoattractant protein 1
miR-200s: miR-200 family
miRNA: MicroRNA
MMP: Metalloproteinase
mRNA: Messenger RNAs
NLS: Nonlesional skin
PASI: Psoriasis Area and Severity Index
Pso: Psoriatic patients
PWV: Pulse wave velocity
ROS: Reactive oxygen species
RWT: LV relative wall thickness
S.E.M.: Standard error mean
SIRT1: Sirtuin1
VSMC: Vascular smooth muscle cell.
